# Supplementary material for: Prolonged Impact of Bisphosphonates and Glucocorticoids on Bone Mechanical Properties
Source: Pharmaceuticals (Basel). 2025 Jan 26;18(2):164. doi: 10.3390/ph18020164 (PMC11858856; doi:10.3390/ph18020164)
Supplement: Supplementary file 1 [file pharmaceuticals-18-00164-s001.zip › pharmaceuticals-3403671-supplementary.pdf]

pharmaceuticals

Supplementary File

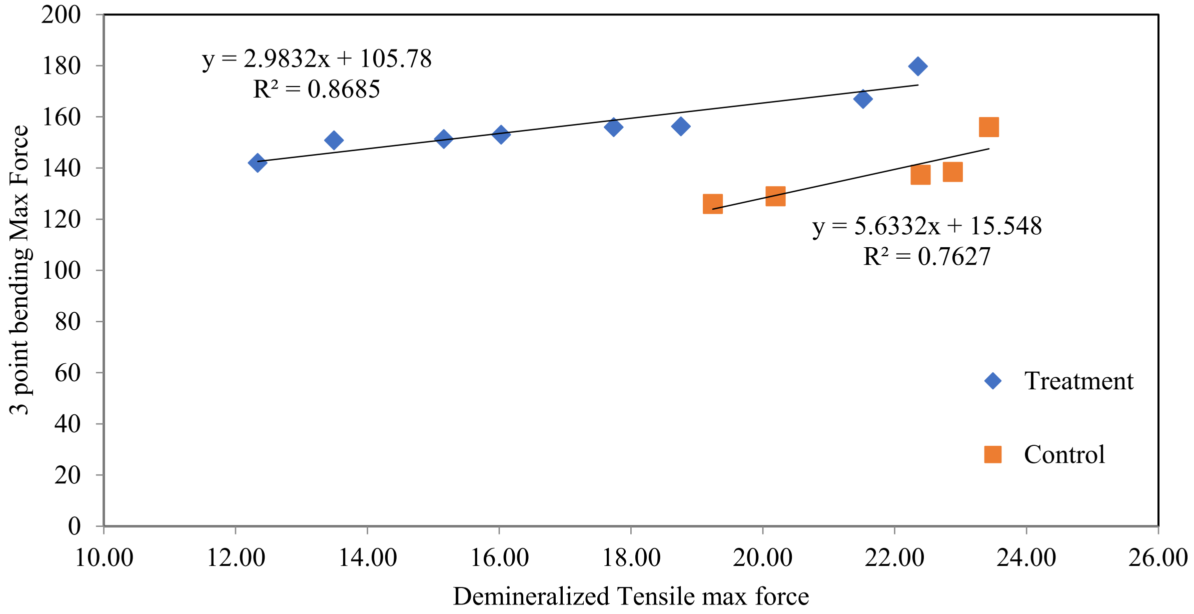

Figure S1:Correlations observed between 3-point bending maximum force and demineralized tensile maximum force for control and treated groups.

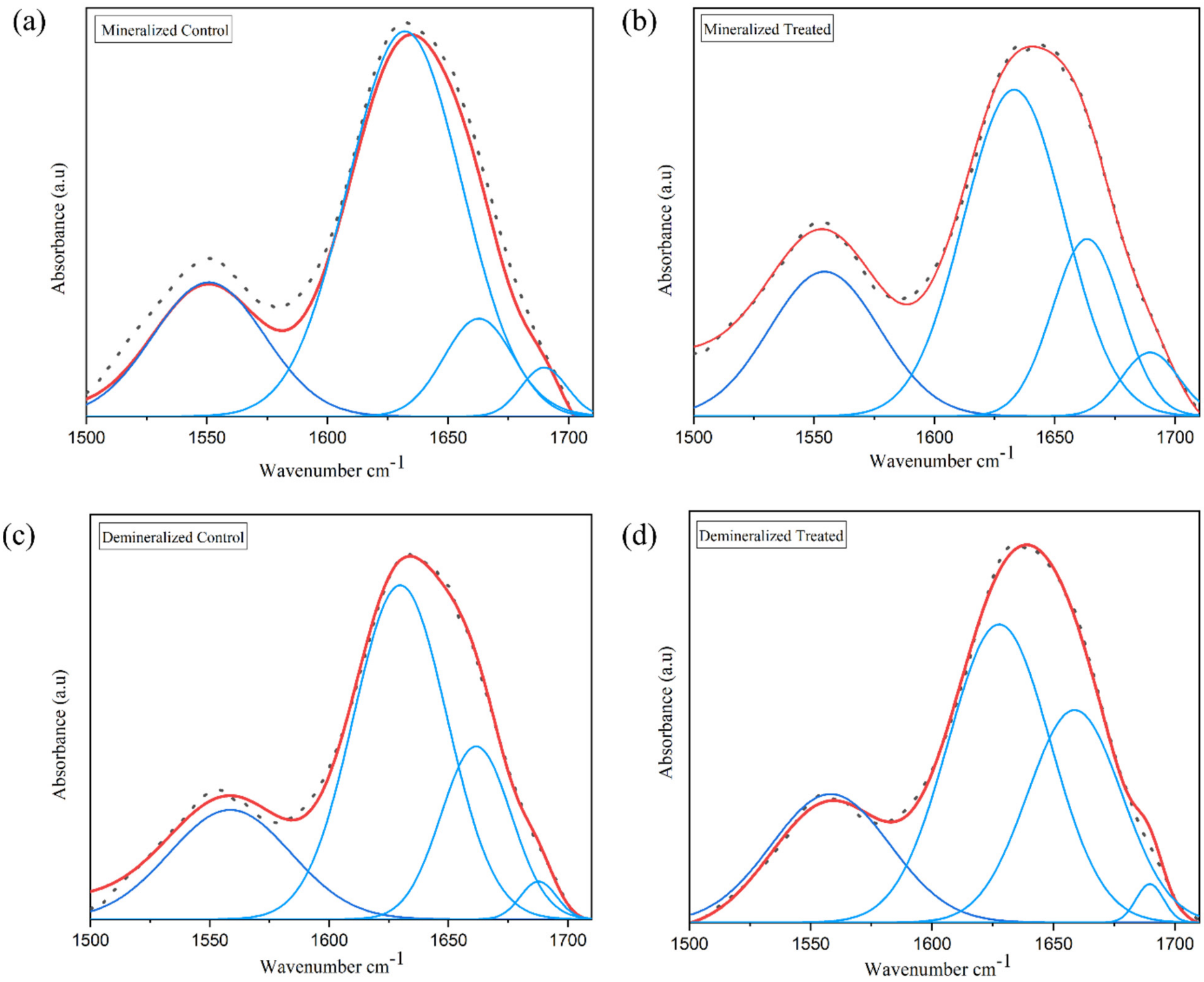

Figure S2: Typical curve fitting analysis of amide I ATR-FTIR band detected in control and treated groups of (a & b) the mineralized and (c & d) the demineralized bone samples.

Table S1: Statistical nonparametric test analysis of ATR-FTIR spectra for treated (T) and control (C) mineralized bone samples.

	Area		FWHM		Center		Height	
Wavelength (cm^{-1})	C	T	C	T	C	T	C	T
1046	63.88 ±14.07	34.68 ±3.81	25.62± 1.85	24.32 ±1.77	1045 ±0.44	1044.42 ±0.00	1.43 ±0.21	0.87 ±0.06
p-value	**0.029***		0.491		0.142		0.081	
1086	32.47 ±5.77	19.18± 1.64	36.60±1. 80	39.72 ±2.99	1086 ±0.42	1085±0.18	0.782±0. 14	0.43 ±0.03
p-value	**0.029***		0.755		0.081		0.059	
1156	8.817±2.2 5	7.17±1.7 8	41.83±6. 91	45.62 ±6.66	1156.5 ±1.11	1155.50 ±0.84	0.211±0. 032	0.156±0. 022
p-value	0.662		1.000		0.491		0.414	
1239	1167±3.04	7.97±1.9 3	818.8±2 35.7	911.7 ±183.92	1239.33 ±0.422	1239.13 ±0.875	0.225 ±0.044	0.155 ±0.025
p-value	0.181		1.000		0.662		0.282	
1273	10.05 ±1.34	7.07 ±.37	42.83 ±2.95	44.12 ±2.51	1273.33 ±.42	1273.25 ±.31	0.26 ±.03	0.17 ±.018
p-value	0.18		0.57		0.85		0.10	
1334	17.04±2.3 7	11.27±0. 94	63.66 ±1.08	63.12 ±1.17	1329.50 ±1.72	1329.50 ±1.99	0.28 ±0.42	0.18 ±0.01

p-value	0.05		0.75		0.14		0.05	
1380	27.79±4.46	17.91±1.45	81.33 ±1.52	83.12 ±1.27	1381.50 ±0.71	1382 ±0.25	0.40 ±0.06	0.24 ±0.02
p-value	0.08		0.28		0.22		0.08	
1451	18.94±3.35	11.35±1.17	56.11 ±2.06	59.20 ±1.26	1453.33 ±0.55	1453.63 ±0.56	0.35 ±0.05	0.22 ±0.02
p-value	0.08		0.28		0.85		0.10	
1546	14.31±6.97	10.07±3.63	300.16 ±240.04	425.25 ±237.39	1545.50 ±1.54	1548.25 ±1.57	0.23 ±0.06	0.16 ±0.04
p-value	0.95		0.85		0.28		0.85	
1647 (amide I)	32.01±5.28	34.56±3.18	95.07 ±5.87	93.73 ±2.00	1644.33 ±2.14	1642.25 ±1.91	0.335 ±0.07	0.34 ±0.03
p-value	**0.02***		1		0.10		0.49	
1740	6.31±1.17	5.14±1.05	38.27 ±2.95	35.8 ±2.58	1742.50 ±0.88	1739.63 ±0.65	0.12 ±0.26	0.08 ±0.01
p-value	0.34		0.75		**0.02***		0.41	
1923	2.61±0.49	1.05±0.17	95.54 ±7.32	98.75 ±6.47	1921.37 ±1.56	1916.50 ±2.04	0.029 ±0.004	0.02 ±0.001
p-value	0.49		0.85		**0.02***		0.66	
2129	5.48±1.71	10.64±0.86	189.77 ±67.63	308.84 ±29.63	2131.83 ±1.30	2139.38 ±11.80	0.029 ±0.002	0.006 ±0.002

p-value	**0.04***		0.14		0.49		0.08	
2894 (CH_2)	43.17±9.6<br>3	21.88±3.<br>02	991.66<br>±583.0	1452.02<br>±523.38	2895.67<br>±1.40	2890.50<br>±5.63	0.339<br>±0.06	0.18<br>±0.01
p-value	**0.02***		0.66		0.57		0.05	
2925	16.37±3.9<br>6	9.50±1.6<br>0	43.83<br>±3.92	46.50<br>±3.39	2926.50<br>±0.67	2927.13<br>±0.85	0.40<br>±0.08	0.21<br>±0.03
p-value	0.22		1		0.34		0.05	
2974	21.62±3.8<br>5	13.49±1.<br>05	43.43<br>±3.97	49.92<br>±3.45	2974.67<br>±0.76	2977.63<br>±0.73	0.52<br>±0.09	0.31<br>±0.02
p-value	0.18		0.28		**0.02***		0.18	
3090	12.24±1.0<br>9	13.93±0.<br>87	2526<br>±493.21	3012.2<br>±1.97	3089.83<br>±0.16	3090.14<br>±0.17	0.21<br>±0.012	0.23<br>±0.009
p-value	0.414		0.573		0.662		0.345	
3323<br>amide A	221.52±8.<br>34	241.52±<br>1.77	351.05<br>±18.47	399.66<br>±5.29	3323.5<br>±9.92	3330.63<br>±10.29	0.634<br>±0.004	0.62<br>±0.001
p-value	**0.02***		**0.04***		0.66		**0.04***	

* Indicates a significant difference ($p < 0.05$)

Table S2: Statistical nonparametric test analysis of ATR-FTIR spectra for treated (T) and control (C) demineralized bone samples.

	Area		FWHM		Center		Height	
Wavelength (cm^{-1})	C	T	C	T	C	T	C	T
1080	31.99 ± 1.60	26.50 ± 1.87	123.11 ± 2.32	109.75 ± 4.68	1080.27 ± 0.54	1077.13 ± 0.90	0.29 ± 0.01	0.26 ±0.12
p-value	**0.029***		**0.013***		**0.001***		0.108	
1160	20.51 ± 2.15	21.03 ± 2.19	72.37 ± 5.2	82.87 ± 5.00	1162.13 ± 0.350	1160.25 ± 0.726	0.3033 ± 0.020	0.168 ± 0.0177
p-value	1.000		0.414		**0.008***		0.081	
1239	31.75 ± 1.76	26.35 ± 1.08	113.50 ± 3.54	108.00 ± 4.60	1239.50 ± 0.267	1238.75 ± 0.313	0.332 ± 0.016	0.284 ± 0.17
p-value	**0.008***		0.852		**0.013***		**0.029***	
1334	11.53 ± 0.821	11.18 ± 0.440	46.50 ± 3.28	50.62 ± 0.323	1340 ± 0.327	1341.75 ± 1.373	0.255 ± 0.009	0.226 ± 0.009
p-value	0.345		0.491		**0.029***		**0.020***	
1380	14.03 ± 1.18	14.08 ± 0.584	55.25 ± 4.51	61.62 ± 1.22	1379.33 ± 0.27	1382.75 ± 3.05	55.25 ± 4.51	3.46 ± 1.22
p-value	0.491		0.346		**0.029***		**0.043***	
1451	17.96	14.39	69.25	62.37	1456	1456.88	0.300	0.257

	± 1.60	± 0.747	± 5.23	± 1.10	± 0.267	± 0.693	± 0.014	± 0.013
p-value	0.345		0.228		0.345		**0.020***	
1546	31.43<br>± 1.72	26.54<br>± 1.70	79.39<br>± 2.54	83.39<br>± 2.19	1552.63<br>± 0.498	1553.06<br>± 0.423	0.453<br>± 0.026	0.382<br>± 0.020
p-value	**0.020***		0.491		1.000		**0.029***	
1647	72.44<br>± 1.27	68.23<br>± 1.38	87.35<br>± 1.35	92.20<br>± 2.15	1633.25<br>± 0.750	1633.38<br>± 0.800	0.807<br>± 0.022	0.732<br>± 0.024
p-value	**0.020***		**0.020***		0.662		**0.043***	
1744	11.55<br>± 1.40	9.77<br>± 0.975	31.63<br>± 2.15	30.13<br>± 1.52	1742.38<br>± 0.263	1742.54<br>± 0.500	0.2247<br>± 0.0031	0.1794<br>± 0.018
p-value	0.228		1.000		0.059		0.108	
2129	22.56<br>± 0.609	22.66<br>± 0.363	452.46<br>± 5.96	453.59<br>± 12.62	2119.75<br>± 3.504	2116.88<br>± 4.414	0.0558<br>± 0.001	0.055<br>± 0.041
p-value	0.282		1.000		0.282		0.345	
2894	14.21<br>± 0.95	12.04<br>± 0.791	38.87<br>± 0.918	35.88<br>± 0.6613	2854.13<br>± 0.227	2854<br>± 0.463	0.180<br>± 0.022	0.150<br>± 0.014
p-value	**0.029***		0.142		0.181		0.108	
2925	20.16<br>± 1.91	16.41<br>± 1.90	77.06<br>± 2.73	70.63<br>± 3.31	2925.50<br>± 0.327	2925.13<br>± 0.789	0.306<br>± 0.034	0.261<br>± 0.023
p-value	0.081		**0.005***		0.228		0.108	
2973	1.81	4.91	379.75	387.50	2976.25	2979.25	0.196	0.0174

	± 0.962	± 1.13	± 370.6	± 365.11	± 0.701	± 1.08	± 0.004	± 0.0061
p-value	**0.005***		**0.043***		**0.043***		0.108	
3311	428.70<br>±1.48	429.23<br>±1.27	414.50<br>±1.99	414.60<br>±1.53	3302.88<br>±4.414	3306.50<br>±3.74	0.997<br>±0.011	0.987<br>±0.020
p-value	0.228		0.181		0.573		0.755	

* Indicates a significant difference ($p < 0.05$)

Table S3: Bone quality parameters from ATR-FTIR spectra analysis.

	Mineralized		Demineralized	
Bone quality parameter	C	T	C	T
Mineral to matrix ratio	5.3280 (± 5.6652)	1.5366 (± 0.5757)	0.0524 (± 0.0120)	0.0519 (± 0.0142)
p-value	0.06148		0.83366	
amide I/amide II ratio	1.9211 (± 0.7321)	2.4084 (± 0.5717)	1.9002 (± 0.0658)	1.8358 (± 0.1148)
p-value	0.4009		0.64552	
